# Determinants of Perceived Treatment Effectiveness in Women with Fibromyalgia: A Cross-Sectional Study

**DOI:** 10.3390/jcm15155956

**Published:** 2026-07-30

**Authors:** Robert Gajda, Marzena Jeżewska-Zychowicz

**Affiliations:** 1 Department of Human Nutrition, Faculty of Biotechnology and Food Sciences, Wrocław University of Environmental and Life Sciences, Chełmońskiego 37, 51-630 Wroclaw, Poland; 2 Department of Food Market and Consumer Research, Institute of Human Nutrition Sciences, Warsaw University of Life Sciences (SGGW-WULS), Nowoursynowska 159C, 02-776 Warsaw, Poland; marzena_jezewska_zychowicz@sggw.edu.pl

**Keywords:** fibromyalgia, quality of life, psychosocial functioning, diet quality, vitamin D, women

## Abstract

**Background**: Fibromyalgia is a chronic pain syndrome in which treatment effectiveness may be influenced by biological, psychological, social, and nutritional factors. This study aimed to identify clinical, psychosocial, nutritional, and selected individual factors associated with patients’ perceived overall treatment effectiveness among women with fibromyalgia. Specifically, we examined whether fibromyalgia symptom severity, psychological and social quality of life, diet quality, vitamin D_3_ supplementation, treatment duration, and selected individual characteristics were associated with patients’ perceptions of treatment effectiveness. **Methods**: A cross-sectional online study included 195 women with physician-diagnosed fibromyalgia who reported using medications and/or supplements. Fibromyalgia severity (FAS, FIQR), quality of life in psychological and social domains (WHOQOL-BREF), diet quality (pHDI, nHDI, DQI), vitamin D_3_ supplementation, and treatment duration were assessed. Cluster analysis and hierarchical linear regression were performed to identify factors associated with self-perceived treatment effects. **Results**: Women reporting lower treatment effects presented greater symptom severity, higher FIQR scores, and lower quality of life in psychological and social domains. No significant differences were observed according to age, BMI, diet quality, or self-reported daily vitamin D_3_ supplementation. In the final regression model, treatment duration (β = 0.246; *p* < 0.001), quality of life in the psychological domain (β = 0.173; *p* = 0.035) and social domain (β = 0.158; *p* = 0.039), and symptom severity (β = −0.196; *p* = 0.041) independently predicted self-perceived treatment effects. **Conclusions**: Better psychological and social well-being, lower symptom severity, and longer treatment duration were associated with higher perceived treatment effectiveness, whereas neither diet quality nor self-reported daily vitamin D_3_ supplementation was associated with this outcome. These findings support a multidisciplinary biopsychosocial model of care integrating nutritional, medical, psychological, and social interventions.

## 1. Introduction

Fibromyalgia is a chronic syndrome characterized by widespread musculoskeletal pain accompanied by sleep disturbances, fatigue, cognitive impairment, and other somatic symptoms [1,2]. Globally, the prevalence of fibromyalgia is estimated to range from approximately 2% to 8% of the population, with the condition occurring around three times more frequently in women than in men [3,4]. Recent studies conducted in the Polish population indicate that the prevalence of fibromyalgia symptoms is comparable to that reported in other European countries [5,6].

Current clinical guidelines and systematic reviews recommend an individualized, multidisciplinary approach combining patient education, physical activity, psychological interventions, and pharmacological treatment as the most effective strategy for fibromyalgia management, whereas pharmacological treatment alone rarely provides satisfactory symptom improvement [2,7]. According to the European Alliance of Associations for Rheumatology (EULAR), regular aerobic exercise, resistance training, and cognitive behavioural therapy constitute the cornerstone of treatment because they improve pain, physical function, and health-related quality of life [8]. Nutritional therapy, particularly the Mediterranean diet and other anti-inflammatory dietary patterns, has emerged as a promising adjunctive strategy [9,10,11]. Similarly, dietary supplementation, including vitamin D, coenzyme Q10, magnesium, tryptophan, probiotics, melatonin, omega-3 fatty acids, and antioxidant compounds, may complement comprehensive management, although the available evidence remains heterogeneous and generally of moderate to low quality [10,12,13,14,15,16]. Patients’ perceptions of treatment outcomes may influence both treatment effectiveness and engagement in the therapeutic process [17,18,19]. Previous studies have demonstrated associations between clinical improvement and patients’ perceptions of treatment outcomes, which may also change over time [20,21]. Moreover, patients’ and physicians’ evaluations of treatment effectiveness and therapeutic goals may differ, highlighting the importance of patient-reported perceptions in clinical decision-making [21]. Patients’ perceptions of treatment outcomes are shaped by multiple factors, including placebo and nocebo effects related to treatment expectations [22] and psychological characteristics such as optimism and anxiety [23,24]. Other clinical and lifestyle-related factors, including age, disease duration, symptom severity, and dietary habits, may also play a role in patients’ perceptions of treatment outcomes; however, their role in women with fibromyalgia has not yet been sufficiently investigated [11,20,21]. Crum and Zuckerman [25] emphasized the importance of understanding patients’ beliefs regarding treatment effectiveness, their potential for modification, and the factors associated with treatment evaluations. The limited evidence available in this area highlights the need for further research to test the following hypotheses: among women with fibromyalgia, perceived treatment effectiveness is negatively associated with symptom severity, body mass index (BMI), and age, whereas it is positively associated with treatment duration, psychological and social domains of quality of life, and dietary quality. Accordingly, the aim of the present study was to identify clinical, psychosocial, nutritional, and selected individual factors associated with patients’ perceived overall treatment effectiveness among women with fibromyalgia. Specifically, we examined whether fibromyalgia symptom severity, psychological and social quality of life, diet quality, vitamin D_3_ supplementation, treatment duration, and selected individual characteristics were associated with patients’ perceptions of treatment effectiveness.

## 2. Materials and Methods

### 2.1. Study Design and Sample

This cross-sectional study was conducted between the second half of March and April 2026 among individuals with fibromyalgia who were members of nationwide Facebook support groups, including Fibromialgia, Razem w Fibromialgii (“Together with Fibromyalgia”), and the Fibro-My Association. Active members of these groups were invited to complete an online questionnaire developed using Google Forms. A total of 236 women responded positively to the invitation and completed the survey. Of these, 36 participants were excluded for the following reasons: lack of informed consent to participate (n = 2), self-report of not having fibromyalgia (n = 3), failure to confirm a physician-established diagnosis of fibromyalgia (n = 10), no current use of medication and/or dietary supplements (n = 6), and incomplete questionnaire data (n = 13). In addition, seven male respondents who had otherwise completed the questionnaire correctly were excluded because of their small number in the study sample. Consequently, the final analysis included 195 questionnaires completed by women with physician-diagnosed fibromyalgia who reported using medication and/or dietary supplements. The study was designed as an exploratory cross-sectional survey using a convenience sample. Therefore, the final sample size was determined by the availability of eligible participants during the recruitment period and included all women who met the predefined inclusion criteria and completed the questionnaire (n = 195). The adequacy of this sample for the planned regression analyses was subsequently confirmed by a post hoc power analysis, as described in Section 2.3.

The study was conducted in accordance with the Declaration of Helsinki. Participation in the survey was voluntary. Informed consent was obtained from all participants. The study was approved by the Rector’s Committee for Research Ethics of the Wrocław University of Environmental and Life Sciences on 16 March 2026, following an opinion on the research project’s compliance with ethical principles (Resolution No. N0N00000.0020.1.3.5.2026).

### 2.2. Questionnaire

The presence and severity of fibromyalgia were assessed using the Modified Fibromyalgia Assessment Scale (FAS 2019 modCr) [26]. This instrument has demonstrated good validity, reliability, and diagnostic agreement with the ACR criteria in European populations and was therefore considered appropriate for assessing fibromyalgia severity in the present study [26]. In accordance with the recommended scoring procedure, the total FAS score (range: 0–39) was calculated, together with separate scores for fatigue and sleep quality (range: 0–20) and pain distribution (range: 0–19). Higher scores indicate greater fibromyalgia severity and greater impairment in the respective domains [26]. In accordance with the instrument, respondents assessed pain distribution experienced during the previous week across 19 body regions [26]. In addition, they completed an analogous assessment referring to an earlier period (at least 3 months previously). Subsequently, an individual FAS Positive Changes score was calculated for each respondent to quantify changes in pain distribution over the preceding 3 months. This score represented the number of body regions in which an improvement was observed, defined as the presence of pain in the earlier assessment and the absence of pain in the corresponding body region during the previous week.

Functional status in individuals with fibromyalgia was assessed using the Revised Fibromyalgia Impact Questionnaire (FIQR) [27], employing the validated Polish version of the instrument (FIQ-Pol) [28]. In accordance with the recommended scoring procedure, the total FIQR score (range: 0–100) was calculated, with higher scores indicating a greater impact of fibromyalgia on the patient’s life [29]. In addition, separate scores were calculated for the three FIQR domains reflecting physical functioning, overall impact of the disease, and symptoms. The maximum possible scores were 30 for the Function domain, 20 for the Overall Impact domain, and 50 for the Symptoms domain.

Psychological and social functioning were assessed using items from the WHOQOL-BREF (World Health Organization Quality of Life–Brief) questionnaire [30]. The validated Polish version of the instrument has demonstrated satisfactory construct validity and good internal consistency across all domains, with Cronbach’s α coefficients ranging from 0.69 to 0.81 [31]. Only the items representing the WHOQOL-BREF Psychological domain (six items) and Social Relationships domain (three items) were included in the present study. Responses were provided on a 5-point Likert scale (1 = very poor, 2 = poor, 3 = neither poor nor good, 4 = good, 5 = very good). Domain scores were calculated by summing responses within each domain for each respondent and then transforming the raw scores to a 20-point scale, allowing comparisons across domains. Higher scores indicated better self-perceived quality of life within the respective domain [30].

Diet quality was assessed based on the daily frequency of consumption of 24 food groups [32], including 10 food groups with a potentially beneficial effect on health (the Pro-Healthy Diet Index; pHDI) and 14 food groups with a potentially adverse effect on health (the Non-Healthy Diet Index; nHDI). Respondents reported their usual frequency of consumption of each food group during the 12 months preceding the survey by selecting one of the following response categories: (1) less than once a month or never; (2) 1–3 times a month; (3) once a week; (4) several times a week; (5) once a day; or (6) several times a day. The responses were subsequently converted to daily consumption frequencies as follows: 0 = less than once a month or never; 0.06 = 1–3 times a month; 0.14 = once a week; 0.5 = several times a week; 1 = once a day; and 2 = several times a day [33].

The duration of pharmacological treatment was assessed using the following question: “*For how long have you been taking medication for fibromyalgia on a daily basis?*” Response options were: (1) less than 1 month; (2) 1 month to 6 months; (3) 6 months to 1 year; and (4) more than 1 year.

The average daily vitamin D_3_ supplementation dose was assessed using two questions: (1) “*What dose of vitamin D_3_ do you take as a supplement?*”, with the dose reported in either international units (IUs) or micrograms (μg), and (2) “*How often do you take vitamin D_3_ supplements?*” Vitamin D_3_ doses reported in micrograms were converted to international units using the conversion factor of 1 μg = 40 IU [34]. Following previously published procedures, supplementation frequency categories were converted into semi-quantitative daily frequency values (times/day), whereby the responses never, once a month or less, several times a month, once a week, several times a week, once a day, and several times a day were assigned values of 0, 0.033, 0.082, 0.143, 0.429, 1.0, and 2.5 times/day, respectively [35,36,37]. The average daily vitamin D_3_ intake was calculated using the following equation: *D_day_ = D_portion_* × *F_day_. D_day_* denotes the average daily vitamin D_3_ dose; *D_portion_* denotes the amount of vitamin D_3_ reported per supplementation occasion; and *F_day_* denotes the supplementation frequency expressed as times/day [36,37]. In addition, vitamin D_3_ supplementation was categorized according to current clinical recommendations for adult supplementation, including those applicable to the Polish population, using the following categories: 0 IU/day, <1000 IU/day, 1000–2000 IU/day, 2001–4000 IU/day, and >4000 IU/day [38,39,40].

The perceived effectiveness of medications and/or dietary supplements was assessed using three questions: “*To what extent do you think that taking medications and/or dietary supplements has: (1) reduced your fatigue; (2) improved the quality and/or duration of your sleep; and (3) improved your mood?*” Responses were recorded on 10-point rating scales ranging from 1 (did not reduce fatigue, did not improve sleep quality and/or duration, and did not improve mood, respectively) to 10 (completely eliminated fatigue, substantially improved sleep quality and/or duration, and substantially improved mood, respectively). Following assessment of the internal consistency of the three-item scale using Cronbach’s alpha (α = 0.780), a Perceived Treatment Effectiveness Index was calculated by summing the scores for the three items. The index ranged from 3 to 30, with higher scores indicating greater perceived effectiveness of the medications and/or dietary supplements. The Perceived Treatment Effectiveness Index was intentionally developed to capture patients’ subjective perceptions of treatment benefit rather than the overall clinical response to therapy. Fatigue, sleep quality, and mood were selected for inclusion in the index because they represent key patient-reported symptoms that substantially affect daily functioning and quality of life in individuals with fibromyalgia and have been recognised by the OMERACT initiative as important outcome domains for the evaluation of treatment effectiveness [41,42]. Pain was assessed separately using validated disease-specific instruments (FAS and FIQR) and was therefore intentionally excluded from the composite index to minimise conceptual overlap between the predictor and outcome measures and to preserve the independence of the constructs being assessed. This approach is consistent with OMERACT recommendations advocating the assessment of individual outcome domains using separate instruments and the avoidance of measurement redundancy when developing core outcome sets [43].

The participants’ characteristics included age, educational attainment (primary, vocational, secondary, or higher education), place of residence (rural area, town with fewer than 100,000 inhabitants, or city with more than 100,000 inhabitants), household composition (living alone; living with a partner; living with family members without a partner; or living with both a partner and family members), employment status (retired or receiving a disability pension; unemployed; employed on a temporary or occasional basis without receiving a pension or disability benefit; employed on a temporary or occasional basis while receiving a pension or disability benefit; or permanently employed), self-rated financial situation (living very modestly or in poverty, living moderately, or living well or very well), as well as self-reported height and body weight. Height and weight data were used to calculate body mass index (BMI) [44].

### 2.3. Statistical Analysis

Descriptive statistics were used to summarize the demographic characteristics of the study sample and the variables included in the analyses.

Responses to the three questions assessing the perceived effectiveness of medications and/or dietary supplements were used in a cluster analysis to identify clusters that were internally homogeneous while differing from one another on these variables. Participants were clustered using the k-means method. The cluster analysis was performed for exploratory descriptive purposes to facilitate the clinical interpretation of participants with different levels of perceived treatment effectiveness. The primary inferential analysis was based on hierarchical linear regression using the continuous treatment effectiveness index. Three distinct clusters were identified according to the perceived effectiveness of medications and/or dietary supplements: Cluster 1—moderate effectiveness (32.8%); Cluster 2—high effectiveness (28.7%); and Cluster 3—low effectiveness (38.5% of the study sample). The three-cluster solution was supported by cluster selection statistics, including the Cubic Clustering Criterion (CCC), pseudo-*F*, pseudo-*T*_2_, and analysis of variance (ANOVA), which compared the mean values of the clustering variables across clusters (Table 1). In addition, post hoc pairwise comparisons of cluster means were performed using the Student–Waller–Duncan *t*-test with the *k*-ratio procedure.

The clusters were profiled using participants’ demographic and socioeconomic characteristics, including age, educational attainment, place of residence, household composition, self-rated financial situation, employment status, and body mass index (BMI). In addition, the clusters were characterized using fibromyalgia-related variables, including FAS Fatigue and Sleep Quality, FAS Pain Distribution, total FAS score, FAS Positive Changes, FIQR Overall Impact, FIQR Function, FIQR Symptoms, total FIQR score, diet quality indices (nHDI, pHDI, and DQI), scores for the WHOQOL-BREF Psychological and Social Relationships domains, and variables related to medication and/or dietary supplement use (daily vitamin D_3_ supplementation dose and duration of pharmacological treatment). Differences in profile characteristics between the clusters were assessed using the chi-square test of independence for categorical variables and the Kruskal–Wallis test for continuous variables.

Hierarchical linear regression was used to identify factors associated with patients’ perceived treatment effectiveness. The Perceived Treatment Effectiveness Index served as the dependent variable. Variables describing the functioning of individuals with fibromyalgia (Model 1), their psychological and social functioning (Model 2), and medication and/or dietary supplement use (Model 3) were entered sequentially as blocks of explanatory variables in the hierarchical regression analysis. Independent variables were entered into the regression model in a theory-driven hierarchical sequence. The first model (Model 1) included variables describing fibromyalgia status (FIQR Function, FIQR Overall Impact, FIQR Symptoms, total FAS score, and FAS Positive Changes). The second model (Model 2) additionally included quality of life in the Social Relationships and Psychological domains. Finally, the third model (Model 3) included variables related to medication and/or dietary supplement use, namely the average daily vitamin D_3_ supplementation dose and the duration of pharmacological treatment. The normality of the residuals was assessed using a P-P plot, which showed residuals closely adhering to the diagonal line. The Shapiro–Wilk test also confirmed that the residuals were normally distributed. The assumptions of linearity and homoscedasticity were confirmed by visual inspection of the scatterplot of standardized residuals against standardized predicted values. Finally, multicollinearity was assessed using Variance Inflation Factors (VIFs) and tolerance values. All VIF values were below 5.0 (ranging from 1.058 to 2.377), and tolerance values were above 0.10 (ranging from 0.421 to 0.945).

A post hoc power analysis was conducted for the final hierarchical regression model in accordance with Cohen’s methodology [45]. Assuming a significance level of α = 0.05, a sample size of 195 participants, nine predictors, and the observed coefficient of determination (R^2^ = 0.213), the statistical power exceeded 0.99, indicating that the study had sufficient power to detect the observed associations. According to Cohen’s criteria, the effect size (*f*^2^ = 0.27), calculated as *R*^2^/(1 − *R*^2^), corresponds to a moderate-to-large effect [45,46].

A two-sided *p*-value of <0.05 was considered statistically significant. All statistical analyses were performed using IBM SPSS Statistics for Windows, Version 31.0 (IBM Corp., Armonk, NY, USA).

### 2.4. Characteristics of the Study Group

The characteristics of the study sample are presented in Table 2. The study included 195 individuals diagnosed with fibromyalgia. The largest proportion of participants were aged 46–55 years (33.3%), followed by those aged 36–45 years (29.2%). Most respondents had completed higher education (61.5%) and resided in cities with more than 100,000 inhabitants (48.7%). The majority were living with a partner, either as a couple (39.5%) or together with both a partner and other family members (33.8%). Nearly half of the participants rated their financial situation as moderate (48.2%), whereas 33.3% reported a good or very good financial situation. Approximately half of the respondents were in permanent employment (50.7%). Based on BMI, 34.4% of participants were classified as overweight and 27.7% as obese (Table 2).

## 3. Results

The identified clusters of individuals with similar evaluations of the effects of medication and/or dietary supplement use did not differ significantly with respect to education, place of residence, marital status, self-perceived financial situation, employment status (Table 1), or body mass index (BMI) and age (Table 1 and Table 3).

The ‘Low effect’ cluster was characterized by the highest median scores for FAS fatigue severity, FIQR Overall Impact, FIQR Symptoms, and FIQR Total. At the same time, the median values of these indicators were lower in the ‘High effect’ cluster than in the ‘Moderate effect’ cluster, except for FAS fatigue severity and sleep quality, for which the median values were identical across the two clusters. The ‘Low effect’ cluster was also characterized by lower median scores in the Psychological and Social Relationships domains of quality of life. No significant differences between the clusters were observed for diet quality indices (nHDI, pHDI, and DQI), duration of medication use, or the daily dose of vitamin D_3_ supplementation (Table 3).

Table 4 presents the results of the hierarchical linear regression analysis examining the association between the medication and/or dietary supplement effectiveness index and disease severity and its impact on the functioning of individuals with fibromyalgia (Model 1), their functioning in the psychological and social domains of quality of life (Model 2), and medication and/or dietary supplement use (Model 3). In Model 1, the FIQR Symptoms score was the only significant predictor of the self-perceived effectiveness of medication and/or dietary supplement use (β = −0.313; *p* < 0.001). After the Psychological and Social Relationships domain scores of quality of life were entered into Model 2, only the Psychological domain score remained positively associated with the self-perceived effectiveness of medication and/or dietary supplement use (β = 0.179; *p* = 0.024). At the same time, the predictive effect of the FIQR Symptoms score was slightly attenuated (β = −0.207; *p* = 0.035).

## 4. Discussion

This study aimed to identify clinical, psychosocial, nutritional, and selected individual factors associated with patients’ perceived overall treatment effectiveness among women with fibromyalgia. Specifically, we examined whether fibromyalgia symptom severity, psychological and social quality of life, diet quality, vitamin D_3_ supplementation, treatment duration, and selected individual characteristics were associated with patients’ perceptions of treatment effectiveness. The findings indicate that a higher perceived treatment effectiveness was associated with better psychological and social quality of life, a longer treatment duration, and lower symptom severity. In contrast, no associations were observed with age, body mass index (BMI), socioeconomic characteristics, diet quality, or the reported daily dose of vitamin D_3_ supplementation.

Fibromyalgia symptom severity emerged as one of the most important factors associated with the perceived effectiveness of treatment, consistent with previous studies demonstrating that persistent fatigue, sleep disturbances, pain, and other disease-related symptoms strongly influence both the perceived effectiveness of therapy and patients’ quality of life [2,7,47,48]. In the regression analysis, the FIQR Symptoms score was the only significant clinical predictor, suggesting that the perceived effectiveness of treatment is more closely related to the current symptom burden than to objective changes in functional status. Although pain is a cardinal symptom of fibromyalgia and has been identified as a determinant of perceived treatment effectiveness [49], the findings of the present study are not unequivocal. Perceived pain as one of several fibromyalgia symptoms, as reflected by the FIQR Symptoms score, was negatively associated with the perceived effectiveness of treatment, whereas the number of painful body regions (FAS pain distribution) showed no such association. Likewise, improvement in this domain (i.e., a reduction in the number of painful body regions following treatment) was not associated with perceived treatment effectiveness. The absence of a clear relationship between the reported distribution of pain and the perceived effectiveness of therapy may indicate that pain intensity, rather than the number of painful body regions, is more relevant to patients’ evaluation of treatment. This interpretation is supported by the pain intensity item included in the FIQR, although it represents only one of several fibromyalgia symptoms assessed by the instrument. These findings are consistent with previous evidence indicating that, in fibromyalgia, patients’ perceptions of treatment outcomes are influenced primarily by the overall burden of symptoms, including pain severity, fatigue, sleep disturbances, and their impact on daily functioning and quality of life, rather than by a single clinical characteristic alone [2,7,47,48,49]. Therefore, a reduction in the number of painful body regions may not necessarily translate into a better subjective evaluation of treatment if pain intensity and the overall symptom burden remain substantial. At the same time, these findings suggest that further research is warranted to determine whether pain intensity is a more clinically meaningful indicator of perceived treatment effectiveness than pain distribution and to further evaluate the usefulness of the FAS as a simple tool for assessing fibromyalgia severity [26].

The duration of treatment was also a significant predictor of perceived treatment effectiveness, with longer treatment duration associated with better treatment outcomes. This finding may reflect the effectiveness of long-term therapy [21]. However, it is also plausible that, with increasing therapeutic experience, patients adapt more effectively to living with a chronic disease, develop more realistic expectations regarding treatment, and demonstrate greater treatment acceptance, all of which may contribute to a more favourable perception of its effectiveness [20,21,25,50].

No differences in perceived treatment effectiveness were observed across diet quality indices (nHDI, pHDI, and DQI). This finding is consistent with previous evidence suggesting that the relationship between diet and fibromyalgia remains inconclusive, with beneficial effects reported mainly in intervention studies evaluating anti-inflammatory or Mediterranean dietary patterns, whereas cross-sectional studies have yielded inconsistent results [9,51,52,53,54,55]. Moreover, the diet quality indices used in the present study provide a general assessment of diet quality rather than its inflammatory potential [9,55].

Similarly, no association was observed between the reported daily dose of vitamin D_3_ supplementation and perceived treatment effectiveness. This finding should be interpreted cautiously because serum 25-hydroxyvitamin D [25(OH)D] concentrations were not measured. Current evidence indicates that vitamin D supplementation may reduce pain severity and improve quality of life primarily in patients with vitamin D deficiency, whereas routine supplementation has not consistently demonstrated benefits in unselected patients with fibromyalgia [12,13,40,56,57,58,59,60]. Accordingly, current guidelines recommend assessing serum 25(OH)D concentrations and correcting confirmed deficiency rather than recommending universal vitamin D supplementation for fibromyalgia [60].

The positive association between psychological and social quality of life and perceived treatment effectiveness supports the biopsychosocial model of fibromyalgia, according to which patients’ perceptions of treatment effectiveness are determined not only by somatic symptoms but also by psychological well-being, social relationships, and adaptive capacity [47,50,61].

The absence of associations between perceived treatment effectiveness and age, body mass index (BMI), or socioeconomic characteristics suggests that these factors make a limited contribution once clinical and psychosocial variables are taken into account [2,47].

The findings supported the hypotheses regarding the associations of perceived treatment effectiveness with symptom severity, treatment duration, and psychological and social quality of life, whereas the hypotheses concerning age, body mass index (BMI), diet quality, and vitamin D_3_ supplementation were not supported. However, the absence of associations with nutritional factors, including overall diet quality and vitamin D_3_ supplementation, should not be interpreted as evidence that these factors have no role in the management of fibromyalgia [12,24,55,62].

A major strength of this study is its comprehensive biopsychosocial approach, which simultaneously assesses fibromyalgia symptom severity, psychological and social quality of life, diet quality, vitamin D_3_ supplementation, and socioeconomic factors in relation to perceived treatment effectiveness. This approach is consistent with the contemporary model of care for patients with fibromyalgia. The credibility of the findings is further strengthened by the use of validated instruments (FIQR, WHOQOL-BREF, and KomPAN) and hierarchical linear regression analysis, which enabled the identification of independent predictors of perceived treatment effectiveness.

This study has several limitations that should be considered when interpreting the findings. First, the cross-sectional study design allows for the identification of associations between variables but does not permit the determination of their direction or causal nature. Consequently, it cannot be established whether better psychosocial functioning leads to a more favourable perception of treatment effectiveness or whether the relationship operates in the opposite direction [63,64].

Second, participants were recruited exclusively through Facebook support groups for individuals with fibromyalgia. Although this recruitment strategy facilitated the inclusion of women from different regions of Poland, it may have introduced selection bias because individuals participating in online support communities are not necessarily representative of the broader population of patients with fibromyalgia [65,66]. They may differ with respect to health literacy, treatment expectations, psychological characteristics, engagement in disease self-management, and possibly disease severity, all of which may influence patients’ perceptions of treatment effectiveness. Consequently, the findings should be interpreted with caution, and their generalizability to the broader population of women with fibromyalgia may be limited.

Another limitation of the study is the use of self-reported data. Information on fibromyalgia symptoms, dietary habits, vitamin D_3_ supplementation, and treatment effectiveness was based on participants’ self-reports and was not objectively verified. Likewise, the diagnosis of fibromyalgia relied on participants’ reports of a previous physician-confirmed diagnosis, which may have increased the risk of recall bias, response bias, and case misclassification [67,68].

The assessment of treatment effectiveness was based on an author-developed index incorporating improvements in fatigue, sleep quality, and mood. Although these domains represent some of the most important symptoms of fibromyalgia, the index did not capture other clinically relevant aspects of treatment response, such as pain intensity, physical functioning, or cognitive symptoms, which are assessed by validated instruments commonly used in fibromyalgia research [27,28,54]. The index’s internal consistency was measured; however, it was not externally validated.

Furthermore, dietary intake was assessed using the KomPAN questionnaire, which measures the frequency of food consumption but does not account for portion sizes or actual nutrient intake [32,33]. Similarly, vitamin D_3_ supplementation was assessed solely on the basis of participants’ self-reported intake, without measurement of serum 25-hydroxyvitamin D [25(OH)D] concentrations, precluding an assessment of actual vitamin D status [13,39,40].

An additional limitation of the present study is that all pharmacological treatments and dietary supplements were analysed as a single exposure category. Because fibromyalgia is managed using heterogeneous pharmacological regimens and various dietary supplements with different mechanisms of action and levels of evidence [69,70,71], participants receiving substantially different therapeutic strategies were analysed together. Furthermore, no detailed information was collected regarding medication classes, dosages, treatment adherence, combination therapies, or the use of individual dietary supplements other than vitamin D_3_. Consequently, the observed associations should be interpreted as reflecting patients’ overall perceptions of their current treatment rather than the effects of specific medications or supplements. Future studies should account for treatment heterogeneity by distinguishing pharmacological classes and supplement types and by including objective measures of treatment adherence and intensity.

Finally, the analysis did not account for several psychological and behavioral factors that may influence perceived treatment effectiveness, including treatment expectations, optimism, depression, anxiety, pain catastrophizing, and placebo and nocebo effects, as well as disease duration, sleep disorders, physical activity level, and detailed characteristics of pharmacological treatment (e.g., medication class, dosage, or combination therapy) [19,22,23,24,50]. This may partly explain why the final regression model accounted for only 21.3% of the variance in perceived treatment effectiveness, suggesting that additional clinical and psychosocial factors not included in the present study also contribute to patients’ perceptions of treatment effectiveness.

## 5. Conclusions

Among women with fibromyalgia, better psychological and social well-being, lower symptom severity, and longer treatment duration correlated with higher self-assessment of treatment effects. No significant association was observed between perceived treatment effectiveness and overall diet quality indices or the self-reported daily dose of vitamin D_3_ supplementation.

In clinical practice, one goal of therapy may be to improve the patient’s psychological and social functioning, as these factors may influence perceptions of treatment effectiveness and engagement in the therapeutic process.

The findings support the implementation of a biopsychosocial model of care for individuals with fibromyalgia, integrating pharmacological treatment with psychological interventions, health education, physical activity, and social support.

No significant association was observed between perceived treatment effectiveness and overall diet quality indices or the self-reported daily dose of vitamin D_3_ supplementation. However, because serum 25-hydroxyvitamin D [25(OH)D] concentrations were not measured, these findings should not be interpreted as evidence that vitamin D status is unrelated to perceived treatment effectiveness. Nutritional interventions should therefore be individualized and incorporated into a comprehensive therapeutic approach rather than implemented as a standalone strategy to improve treatment outcomes.

From a clinical nutrition perspective, the findings support the need to integrate nutritional counselling with the assessment of patients’ psychosocial functioning. Such an approach may facilitate more effective implementation of therapeutic recommendations and improve the long-term treatment outcomes of women with fibromyalgia.

The lack of associations with dietary quality indices suggests that future research should focus more on dietary patterns with proven anti-inflammatory potential, on objectively assessed vitamin D levels, and on their associations with patient-perceived treatment outcomes and clinical indicators.

## Figures and Tables

**Table 1:** Characteristics of the Identified Clusters According to Self-Reported Effects of Medication and/or Supplement Use (Mean Values and Standard Deviations) *.

Effects of Medication and/or Supplement Use in Relation to:	Cluster			*p*-Value
	1 Moderate Effect	2 Large Effect	3 Small Effect	
Fatigue	3.8 a ** (1.61)	6.3 b (1.85)	1.8 c (0.94)	<0.001
Sleep quality and/or duration	4.3 a (1.86)	8.0 b (1.51)	2.9 c (2.20)	<0.001
Mood	5.6 a (1.81)	7.0 b (1.76)	1.9 c (0.92)	<0.001

* 10-point scale, where 1 = no effect and 10 = substantial positive effect. ** Different letters indicate statistically significant differences between means according to the ANOVA post hoc Waller–Duncan test.

**Table 2:** Characteristics of the Study Group.

Characteristics		Total Sample % (N *)	Cluster			*p*-Value
			1 Moderate Effect	2 Large Effect	3 Small Effect	
Total		100.0 (195)	32.8 (64)	28.7 (56)	38.5 (75)	
Age	35 years and below	19.0 (37)	15.6 (10	16.1 (9)	24.0 (18)	0.101
	36–45 years	29.2 (57)	35.9 (23)	37.5 (21)	17.3 (13)	
	46–55 years	33.3 (65)	31.3 (20)	25.0 (14)	41.3 (31)	
	Above 55 years	18.5 (36)	17.2 (11)	21.4 (12)	33.4 (65)	
Education	Primary or vocational	6.6 (13)	4.7 (3)	5.4 (3)	9.3 (7)	0.488
	Secondary	31.9 (62)	34.4 (22)	25.0 (14)	34.7 (26)	
	Higher	61.5 (120)	60.9 (39)	69.6 (39)	56.0 (42)	
Place of residence	Rural area	26.2 (51)	31.3 (20)	17.9 (10)	28.0 (21)	0.534
	A town with fewer than 100,000 inhabitants	25.1 (49)	25.0 (16)	26.8 (15)	24.0 (18)	
	City with more than 100,000 inhabitants	48.7 (95)	43.7 (28)	55.3 (31)	48.0 (36)	
Family status	I live alone	13.9 (27)	17.2 (11)	12.5 (7)	12.0 (9)	0.754
	I live with my partner	39.5 (77)	35.9 (23)	48.2 (27)	36.0 (27)	
	I live with my family, without my partner	12.8 (25)	12.5 (8)	10.7 (6)	14.7 (11)	
	I live with my partner and my family	33.8 (66)	34.4 (22)	28.6 (16)	37.3 (28)	
Self-reported financial situation	I live very modestly or in poverty	18.5 (36)	17.2 (11)	10.7 (6)	25.3 (19)	0.278
	I live moderately	48.2 (94)	51.5 (33)	50.0 (28)	44.0 (33)	
	I live well or very well	33.3 (65)	31.3 (20)	39.3 (22)	30.7 (23)	
Employment Status	I am retired or receiving a disability pension	13.8 (27)	15.6 10)	10.7 (6)	14.7 (11)	0.887
	I am unemployed	26.2 (51)	21.9 (14)	26.8 (15)	29.3 (22)	
	I am employed on a temporary or occasional basis and do not receive a pension or disability benefit	7.2 (14)	4.7 (3)	7.1 (4)	9.3 (7)	
	I am employed on a temporary or occasional basis, but receive a pension or disability benefit	2.1 (4)	3.1 (2)	1.8 (1)	1.3 (1)	
	I have permanent employment	50.7 (99)	54.7 (35)	53.6 (30)	45.3 (34)	
BMI	Underweight	2.1 (4)	0.0 (0)	3.6 (2)	2.7 (2)	0.564
	Normal weight	35.9 (70)	37.5 (24)	32.1 (18)	37.3 (28)	
	Overweight	34.4 (67)	40.6 (26)	33.9 (19)	29.3 (22)	
	Obesity	27.7 (54)	21.9 (14)	30.4 (17)	30.7 (23)	

* N—number of respondents.

**Table 3:** Characteristics of the Identified Clusters According to Selected Variables Describing the Study Population.

Characteristics	Statistical Parameters	Total	Cluster			*p*-Value
			1 Moderate Effect	2 Large Effect	3 Small Effect	
Age	Mean rank		96.6	98.9	98.5	0.970
	Median	46.0	45.0	45.0	48.0	
	IQR	15.0	15.0	16.0	17.0	
	Min–max	19.0–75.0	19.0–66.0	23.0–75.0	19.0–68.0	
BMI	Mean rank		92.9	101.9	99.4	0.656
	Median	26.2	26.0	27.3	26.2	
	IQR	7.2	6.0	8.0	8.5	
	Min–max	17.3–46.2	19.3–44.0	17.5–46.2	17.3–45.9	
FAS—fatigue severity and sleep quality	Mean rank		99.3	81.8	109.0	0.023
	Median	15.0	15.0	15.0	16.0	
	IQR	5.0	5.0	5.0	4.0	
	Min–max.	7.0–20.0	9.0–20.0	7.0–20.0	8.0–20.0	
FAS—pain distribution	Mean rank		101.8	88.6	109.0	0.332
	Median	10.0	11.0	9.0	10.0	
	IQR	7.0	6.0	8.0	7.0	
	Min–max	0.0–19.0	0.0–19.0	0.0–19.0	0.0–19.0	
FAS	Mean rank		102.6	83.6	104.8	0.074
	Median	25.0	26.0	24.0	27.0	
	IQR	8.0	6.0	10.0	10.0	
	Min–max	10.0–39.0	10.0–35.0	12.0–36.0	11.0–39.0	
FAS—positive changes in pain distribution over the past 3 months	Mean rank		102.8	94.9	96.2	0.690
	Median	2.0	2.0	2.0	1.0	
	IQR	5.0	6.0	4.0	5.0	
	Min–max	0.0–19.0	0.0–16.0	0.0–10.0	0.0–19.0	
FIQR Overall Impact	Mean rank		97.7	79.6	112.0	0.005
	Median	16.0	16.0	14.5	17.0	
	IQR	6.0	5.0	7.0	5.0	
	Min–max	3.0–20.0	6.0–20.0	4.0–20.0	3.0–20.0	
FIQR Functioning	Mean rank		98.4	87.2	105.8	0.176
	Median	21.3	21.0	20.3	22.3	
	IQR	7.3	6.8	9.7	7.0	
	Min–max	5.3–28.7	5.3–28.7	6.3–28.7	6.0–28.7	
FIQR Symptoms	Mean rank		93.3	82.4	113.6	0.005
	Median	36.5	36.0	33.5	37.5	
	IQR	9.0	7.9	11.4	11.5	
	Min–max	15.5–49.0	25.0–45.0	15.5–46.5	15.5–49.0	
FIQR Total	Mean rank		96.1	82.0	111.6	0.011
	Median	72.0	71.8	69.2	74.7	
	IQR	18.7	16.8	25.3	20.7	
	Min–max	26.8–97.7	38.3–89.7	26.8–94.8	32.8–97.7	
nHDI	Mean rank		91.4	102.2	100.5	0.515
	Median	9.6	9.3	9.9	9.9	
	IQR	9.2	9.0	14.6	8.2	
	Min–max	0.0–34.1	0.2–31.0	0.4–34.1	0.0–25.7	
pHDI	Mean rank		98.1	99.2	97.0	0.977
	Median	21.7	21.8	21.9	21.2	
	IQR	14.5	17.2	13.7	13.8	
	Min–max	0.6–58.4	0.6–51.3	2.1–47.5	1.6–58.4	
DQI	Mean rank		101.7	92.1	99.3	0.628
	Median	10.4	12.3	8.9	12.7	
	IQR	0.2	18.9	17.5	17.0	
	Min–max	−17.0–55.0	−15.0–40.3	−13.7–35.3	−17.0–55.0	
Psychological Domain of Quality of Life	Mean rank		100.1	118.2	81.1	<0.001
	Median	11.0	11.0	11.0	10.0	
	IQR	3.0	3.0	3.0	2.0	
	Min–max	5.0–15.0	6.0–14.0	6.0–15.0	5.0–15.0	
Social Domain of Quality of Life	Mean rank		98.8	115.0	84.7	0.009
	Median	11.0	11.0	11.5	9.0	
	IQR	5.0	5.0	6.0	4.0	
	Min–max	4.0–20.0	4.0–19.0	5.0–20.0	4.0–16.0	
Daily vitamin D_3_ supplementation dose	Mean rank		101.9	102.0	91.7	0.440
	Median	4000.0	2860.0	4000.0	4000.0	
	IQR	3010	2000.0	2570.0	3428	
	Min–max	0.0–50,000.0	66.0–50,000.0	0.0–12,000.0	0.0–40,000.0	
Duration of medication use	Mean rank		102.5	105.1	88.8	0.081
	Median	4.0	4.0	4.0	4.0	
	IQR	1.0	1.0	0.0	2.0	
	Min–max	0.0–4.0	0.0–4.0	0.0–4.0	0.0–4.0	

Kruskal–Wallis test; IQR, interquartile range.

**Table 4:** Association Between Self-Reported Effects of Medication and/or Supplement Use and Selected Variables (Hierarchical Regression Models).

	B	SE	β (Beta)	*t*	*p*
Model 1—F(5:189) = 6.024; *p* < 0.001; R^2^_adj._ = 0.115					
FIQR Functioning	0.211	0.123	0.177	1.710	0.089
FIQR Overall Impact	−0.252	0.168	−0.153	−1.498	0.136
FIQR Symptoms	−0.303	0.090	−0.313	−3.354	<0.001
FAS	−0.098	0.091	−0.090	−1.080	0.281
FAS—Positive Changes in Pain Distribution Over the Past 3 Months (Fewer Painful Body Areas)	−0.104	0.132	−0.060	−0.788	0.431
Model 2—F(7:187) = 6.222; *p* < 0.001; R^2^_adj._ = 0.159					
FIQR Functioning	0.212	0.121	0.178	1.749	0.082
FIQR Overall Impact	−0.204	0.164	−0.124	−1.240	0.217
FIQR Symptoms	−0.201	0.095	−0.207	−2.120	0.035
FAS	−0.099	0.089	−0.091	−1.113	0.267
FAS—Positive Changes in Pain Distribution Over the Past 3 Months (Fewer Painful Body Areas)	−0.071	0.129	−0.040	−0.547	0.585
Social quality of life	0.282	0.192	0.121	1.469	0.144
Psychological quality of life	0.503	0.220	0.179	2.283	0.024
Model 3—F(9:185) = 6.818; *p* < 0.001; R^2^_adj._ = 0.213					
FIQR Functioning	0.225	0.117	0.189	1.920	0.056
FIQR Overall Impact	−0.213	0.160	−0.130	−1.331	0.185
FIQR Symptoms	−0.190	0.092	−0.196	−2.053	0.041
FAS	−0.131	0.087	−0.120	−1.508	0.133
FAS—Positive Changes in Pain Distribution Over the Past 3 Months (Fewer Painful Body Areas)	−0.034	0.125	−0.020	−0.273	0.785
Social quality of life	0.445	0.214	0.158	2.079	0.039
Psychological quality of life	0.402	0.189	0.173	2.123	0.035
Daily vitamin D_3_ supplementation dose	−0.009	0.000	−0.073	−1.110	0.268
Duration of medication use	1.336	0.357	0.246	3.743	<0.001

F—ANOVA test statistic; Adjusted R^2^—adjusted coefficient of determination; B—unstandardised regression coefficient; SE—standard error; β (Beta)—standardised regression coefficient; *t*—Student’s *t*-test statistic Model 3, additionally adjusted for the duration of medication use and the daily dose of vitamin D_3_ supplementation, explained the greatest proportion of the variance (adjusted R^2^ = 0.213). In this model, significant predictors of the medication and/or dietary supplement effectiveness index were the duration of medication use (β = 0.246; *p* < 0.001), the Psychological domain of quality of life (β = 0.173; *p* = 0.035), the Social Relationships domain of quality of life (β = 0.158; *p* = 0.039), and the FIQR Symptoms score (β = −0.196; *p* = 0.041) (Table 4).

## Data Availability

The research data are available in the Knowledge Base Repository of the Wrocław University of Environmental and Life Sciences at https://bazawiedzy.upwr.edu.pl/info/researchdata/UPWRcbe0b3d7e06e4e5cbf292fc7700fce80/ URL (accessed on 20 May 2026) DOI: 10.57755/48gw-d684.
